# Causal effects of serum lipid biomarkers on early age-related macular degeneration using Mendelian randomization

**DOI:** 10.1186/s12263-023-00730-5

**Published:** 2023-07-21

**Authors:** Fen-Fen Li, Yuqin Wang, Lishuang Chen, Chong Chen, Qi Chen, Lue Xiang, Feng-Qin Rao, Li-Jun Shen, Qin-Xiang Zheng, Quanyong Yi, Xiu-Feng Huang

**Affiliations:** 1 National Clinical Research Center for Ocular Diseases, Eye Hospital, Wenzhou Medical University, Wenzhou, China; 2 State Key Laboratory of Ophthalmology, Optometry and Visual Science, Eye Hospital, Wenzhou Medical University, Wenzhou, China; 3 The Ningbo Eye Hospital, Wenzhou Medical University, Ningbo, China; 4 School of Pharmaceutical Sciences of Wenzhou Medical University, Wenzhou, China; 5 Center for Rehabilitation Medicine, Department of Ophthalmology, Zhejiang Provincial People’s Hospital,Affiliated People’s Hospital, Hangzhou Medical College, Hangzhou, China; 6 Zhejiang Provincial Clinical Research Center for Pediatric Disease, The Second Affiliated Hospital and Yuying Children’s Hospital of Wenzhou Medical University, Wenzhou, China

**Keywords:** Serum lipid biomarkers, Early age-related macular degeneration, Mendelian randomization

## Abstract

**Background:**

Age-related macular degeneration (AMD) is one of the major causes of vision loss. Early AMD needs to be taken seriously, but the causal effects of lipid biomarkers on early AMD remain unclear.

**Methods:**

In this study, two-sample Mendelian randomization (MR) analysis was performed to systematically assess the causal relationships between seven serum lipid biomarkers (apolipoprotein A (ApoA), apolipoprotein B (ApoB), total cholesterol (CHOL), high-density lipoprotein cholesterol (HDL-C), direct low-density lipoprotein cholesterol (LDL-C), lipoprotein A [Lp(a)], and triglycerides (TG)) and risk of early AMD. In total, 14,034 cases and 91,214 controls of European ancestry were included in the analysis (number of SNPs = 11,304,110).

**Results:**

MR estimates revealed that a higher HDL-C level is strongly associated with increased risk of early AMD (*OR* = 1.25, 95% *CI*: 1.15–1.35, *P* = 2.61 × 10^−8^). In addition, level of ApoA is also positively associated with risk of early AMD (*OR* = 2.04, 95% *CI*: 1.50–2.77, *P* = 6.27 × 10^−6^). Conversely, higher levels of TG significantly decrease the risk of early AMD (*OR* = 0.77, 95% *CI*: 0.71–0.84, *P* = 5.02 × 10^−10^). Sensitivity analyses further supported these associations. Moreover, multivariable MR analyses, adjusted for the effects of correlated lipid biomarkers, yielded similar results.

**Conclusion:**

This study identifies causal relationships between elevated circulating HDL-C/ApoA levels and increased risk of early AMD, in addition to finding that TG specifically reduces the risk of early AMD. These findings contribute to a better understanding of the role of lipid metabolism in drusen formation, particularly in early AMD development.

**Supplementary Information:**

The online version contains supplementary material available at 10.1186/s12263-023-00730-5.

## Introduction

Age-related macular degeneration (AMD) is one of the major causes of irreversible visual impairment and central vision loss in individuals over the age of 50 [1]. The prevalence of AMD corresponds to approximately 8.7% of the global population and has been predicted to reach around 200 million by 2020 and nearly 288 million by 2040 [2], posing a heavy public health burden [3]. The causes of AMD are complex, involving environmental and genetic factors that influence susceptibility to its development [1]. The characteristic lesions of AMD are drusen, which are formed by deposits of extracellular debris between the retinal pigment epithelium and Bruch’s membrane [4, 5]. Currently, the progression of AMD is classified into early, intermediate, and late stages based on the severity of fundus lesions, such as drusen size and pigmentary abnormalities [6]. The pathological hallmark of early AMD is the presence of medium-size drusen (≥ 63 µm and < 125 µm) without pigmentary abnormalities [6], which are often asymptomatic and easily ignored in the clinic. Intermediate AMD features drusen of diameter ≥ 125 µm or pigmentary abnormalities [6]. Progression to late AMD is characterized by severe central vision loss caused by either neovascular AMD (nAMD) or geographic atrophy (GA). However, there are no effective medications for the GA subtype, the most prevalent subtype of late AMD, and the anti-vascular endothelial growth factor (anti-VEGF) drugs used to treat nAMD are not curative [7]. Therefore, early screening and prompt treatment are essential to maximize the likelihood of retaining functional vision.

The mechanisms of drusen initiation and formation are not yet fully understood. Preclinical studies have identified drusen-related components, such as lipids and Osteopontin [8, 9]. Lipid metabolism has long been believed to be involved in AMD pathogenesis [10–12]; however, the evidence is inconsistent [13]. These inconsistent results may be due to the effects of nutrition and drugs, which are difficult to control for inpatient populations [14]. In recent years, Mendelian randomization (MR) has emerged as a research method for investigating putative causal relationships between risk factors and diseases by using genetic variants as natural experiments [15]. Compared to traditional observational studies, MR is less likely to be influenced by confounding factors or reverse causation [15, 16]. Existing MR studies have reported a causal relationship between higher levels of high-density lipoprotein cholesterol (HDL-C) and intermediate/advanced AMD risk [17–19]; however, a consensus has yet to be reached regarding the associations between other lipid subfractions (e.g., LDL cholesterol, and TG) and AMD. Some researchers have speculated that these findings point to a role for retina-specific mechanisms, including local lipid trafficking, in AMD pathogenesis [20, 21].

There is a paucity of up-to-date and comprehensive information focused exclusively on early AMD. Crucially, a recent meta-GWAS on early AMD included 11 sources with GWAS data and fundus photography for early AMD phenotyping, representing the largest study of early AMD study to date [22]. More importantly, this study demonstrated the shared and distinct genetics between early and advanced AMD [22]. In this study, we systematically investigated the associations between seven major serum lipid biomarkers [ApoA, ApoB, CHOL, HDL-C, LDL-C, Lp(a), and TG] and risk of early AMD using MR analysis. Elucidating these relationships will help us better understand the role of lipids in early-stage AMD and identify lipid-modifying therapeutic targets.

## Results

### Associations of serum lipid biomarkers with early AMD

**Fig. 1:**
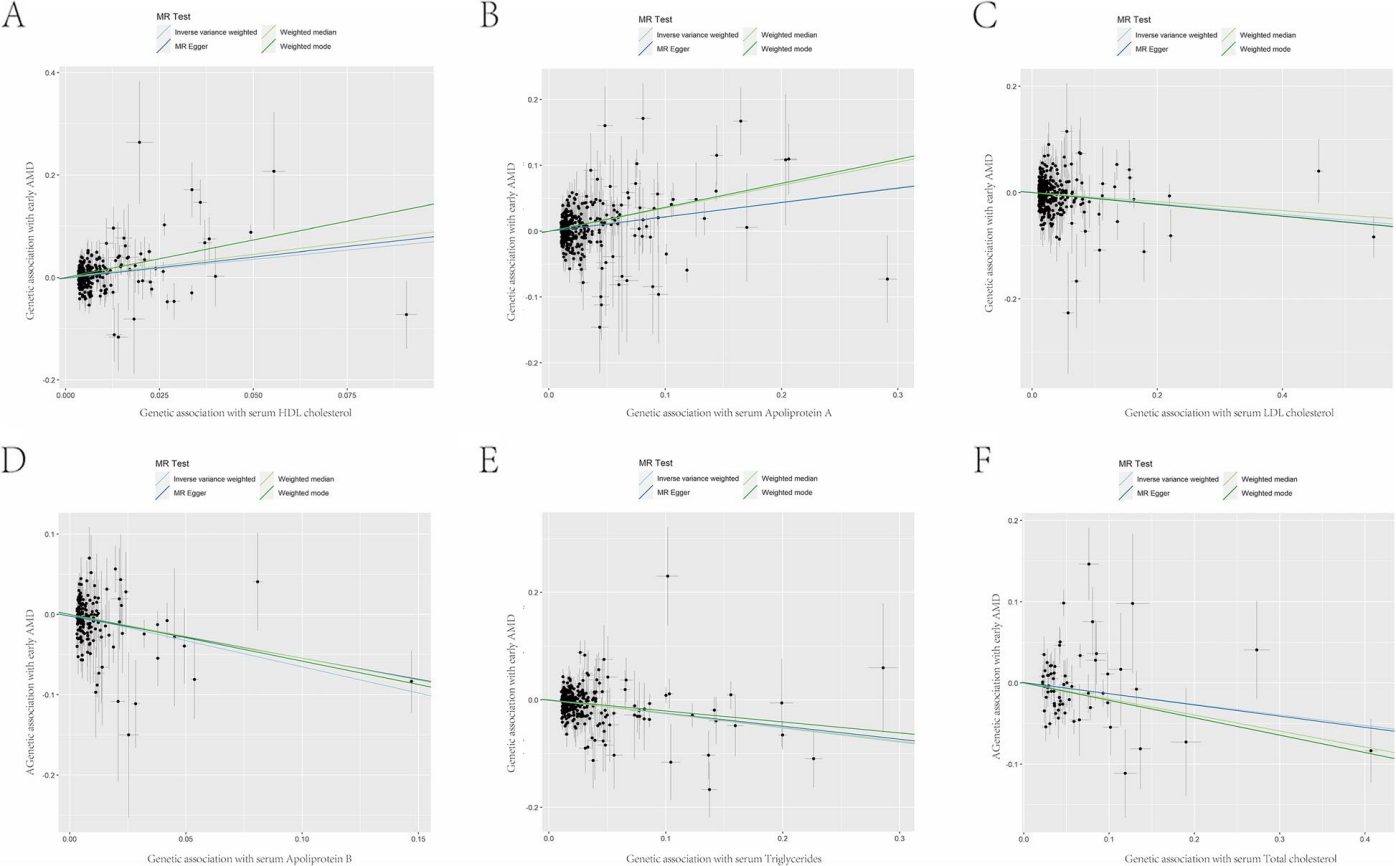
Causal relationships between serum lipid biomarkers and risk of early AMD. Causal relationships between serum HDL-C (**A**), ApoA (**B**), LDL-C (**C**), ApoB (**D**), TG (**E**), and CHOL (**F**) and risk of early AMD. The *x*-axis shows estimated values for serum lipid levels, and the *y*-axis shows estimated (log odd ratios) effects on early AMD. MR-IVW, MR-Egger, simple median, and weighted median method lines are plotted with different colors

**Fig. 2:**
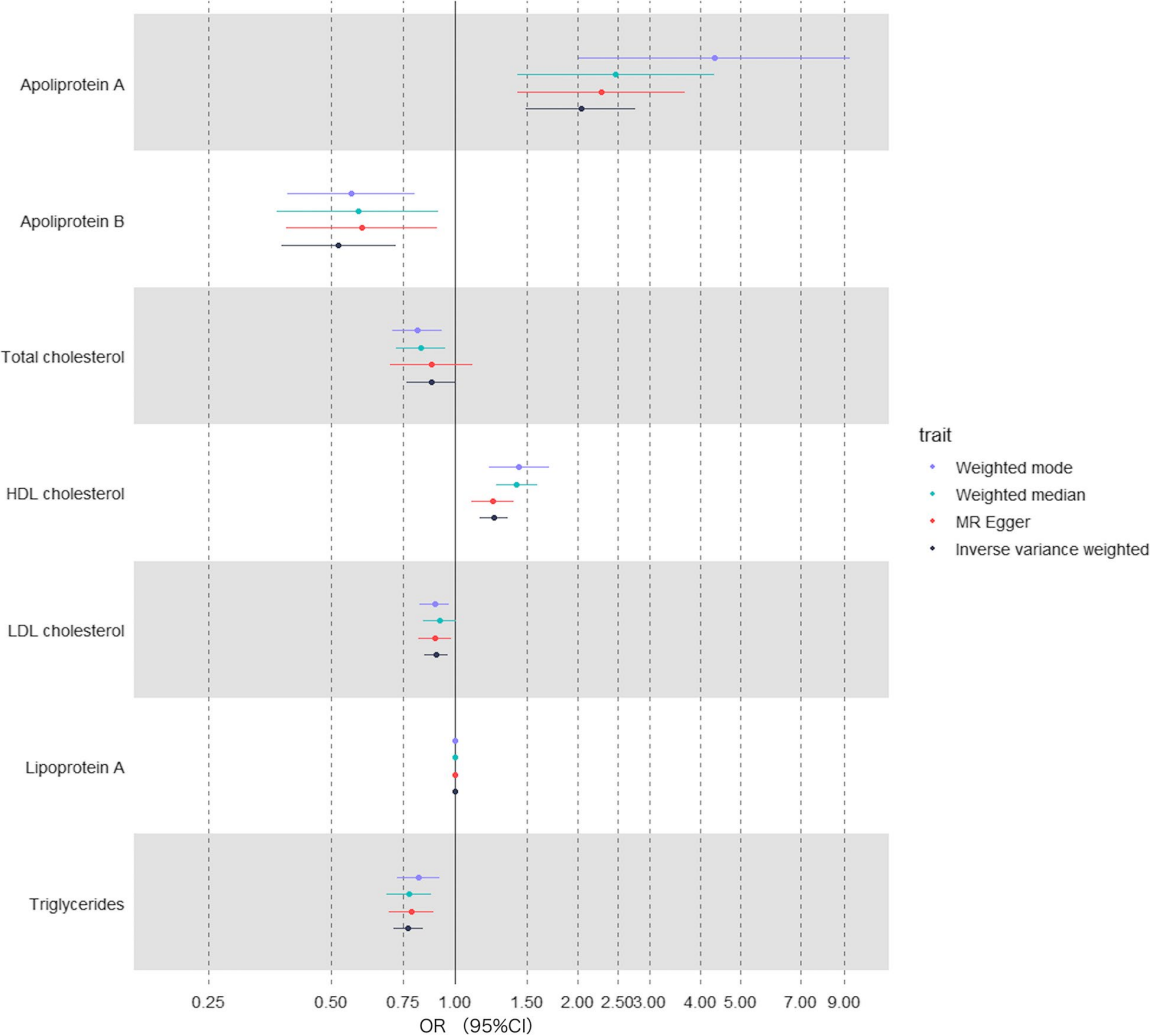
Univariable MR estimates of associations between seven serum lipid biomarkers and early AMD. The vertical line is the reference at *OR* = 1. Different MR methods are displayed with different colors

Of the seven lipid-related traits, univariable MR analysis using the IVW method found that five biomarkers were significantly associated with early AMD, including HDL-C, LDL-C, TG, ApoA, and ApoB (Table 1). MR estimates showed that higher HDL-C levels were strongly associated with increased risk of early AMD (*OR* = 1.25, 95% *CI*: 1.15–1.35, *P* = 2.61 × 10^−8^) (Figs. 1A and 2). Consistent results were found by MR analysis using three other MR methods, including weighted median (*OR* = 1.42, *P* = 4.51 × 10^−9^), weighted mode (*OR* = 1.44, *P* = 2.61 × 10^−5^), and MR Egger (*OR* = 1.24, *P* = 4.32 × 10^−4^). In addition, levels of ApoA were also found to be positively associated with risk of early AMD (*OR* = 2.04, 95% *CI*: 1.50–2.77, *P* = 6.27 × 10^−6^) (Figs. 1B and 2), and these estimates were supported by the other three MR methods (Table 1). These results are consistent with the high correlation between HDL-C and ApoA.

**Table 1:** Casual effect of lipid biomarkers on early AMD

Exposures	SNPs	Methods	Estimate	Standard error	*p*-value	OR	95% *CI*
		**Inverse variance weighted**	**0.71**	**0.16**	**6.27 × 10**^−**6**^	**2.04**	**1.50–2.77**
**ApoA**		**Weighted median**	**0.91**	**0.28**	**1.35 × 10**^−**3**^	**2.48**	**1.42–4.32**
g/L	237	**Weighted mode**	**1.46**	**0.39**	**2.07 × 10**^−**4**^	**4.33**	**2.02–9.27**
		**MR Egger**	**0.83**	**0.24**	**7.15 × 10**^−**4**^	**2.28**	**1.42–3.66**
		Intercept	− 1.41 × 10^−3^	2.25 × 10^−3^	5.33 × 10^−1^		
		**Inverse variance weighted**	− **0.65**	**0.16**	**6.60 × 10**^−**5**^	**0.52**	**0.38–0.72**
**ApoB**		Weighted median	− 0.54	0.23	1.84 × 10^−2^	0.58	0.37–0.91
g/L	166	**Weighted mode**	− **0.58**	**0.18**	**1.85 × 10**^−**3**^	**0.56**	**0.39–0.80**
		MR Egger	− 0.52	0.22	1.67 × 10^−2^	0.59	0.39–0.91
		Intercept	− 2.06 × 10^−3^	2.28 × 10^−3^	3.69 × 10^−1^		
		Inverse variance weighted	− 0.13	0.07	6.90 × 10^−2^	0.87	0.76–1.01
**CHOL**		Weighted median	− 0.19	0.07	8.54 × 10^−3^	0.82	0.70–0.95
mmol/L	63	**Weighted mode**	− **0.21**	**0.07**	**4.09 × 10**^−**3**^	**0.80**	**0.70–0.92**
		MR Egger	− 0.13	0.12	2.60 × 10^−1^	0.87	0.68–1.10
		Intercept	5.11 × 10^−4^	6.63 × 10^−3^	9.38 × 10^−1^		
		**Inverse variance weighted**	**0.22**	**0.04**	**2.61 × 10**^−**8**^	**1.25**	**1.15–1.35**
**HDL-C**		**Weighted median**	**0.35**	**0.06**	**4.51 × 10**^−**9**^	**1.42**	**1.26–1.59**
mmol/L	344	**Weighted mode**	**0.36**	**0.09**	**2.61 × 10**^−**5**^	**1.44**	**1.22–1.70**
		**MR Egger**	**0.22**	**0.06**	**4.32 × 10**^−**4**^	**1.24**	**1.10–1.40**
		Intercept	1.73 × 10^−4^	1.80 × 10^−3^	9.23 × 10^−1^		
		**Inverse variance weighted**	− **0.10**	**0.03**	**2.03 × 10**^−**3**^	**0.90**	**0.85–0.96**
**LDL-C**		Weighted median	− 0.08	0.05	8.02 × 10^−2^	0.92	0.84–1.01
mmol/L	350	Weighted mode	− 0.11	0.04	9.34 × 10^−3^	0.90	0.82–0.97
		MR Egger	− 0.11	0.05	1.68 × 10^−2^	0.90	0.82–0.98
		Intercept	4.04 × 10^−4^	1.66 × 10^−3^	8.08 × 10^−1^		
		Inverse variance weighted	− 1.10 × 10^−4^	5.43 × 10^−4^	8.39 × 10^−1^	1	0.999–1.001
		Weighted median	2.06 × 10^−5^	6.39 × 10^−4^	9.74 × 10^−1^	1	0.999–1.001
**Lp(a)**	19	Weighted mode	− 3.76 × 10^−6^	5.92 × 10^−4^	9.95 × 10^−1^	1	0.999–1.001
nmol/L		MR Egger	7.27 × 10^−4^	7.91 × 10^−4^	3.71 × 10^−1^	1.001	0.999–1.002
		Intercept	− 1.73 × 10^−2^	1.22 × 10^−2^	1.73 × 10^−1^		
		**Inverse variance weighted**	− **0.26**	**0.04**	**5.02 × 10**^−**10**^	**0.77**	**0.71–0.84**
		**Weighted median**	− **0.25**	**0.06**	**8.30 × 10**^−**5**^	**0.78**	**0.68–0.88**
**TG**	295	**Weighted mode**	− **0.20**	**0.06**	**7.30 × 10**^−**4**^	**0.82**	**0.73–0.92**
mmol/L		**MR Egger**	− **0.24**	**0.06**	**2.14 × 10**^−**4**^	**0.79**	**0.69–0.89**
		Intercept	− 8.18 × 10^−4^	1.91 × 10^−3^	6.69 × 10^−1^		

*AMD* Age-related macular degeneration, *CI* Confidence interval, *MR* Mendelian randomization, *OR* odds ratio

*P*-value < 0.0071 considered as significant at genome-wide association level and with bolded numbers

Conversely, we found that higher LDL-C levels significantly decreased risk of early AMD (*OR* = 0.90, 95% *CI*: 0.85–0.96, *P* = 2.03 × 10^−3^) (Figs. 1C and 2). However, the estimates lost significance after Bonferroni correction in the other three MR methods (Table 1). In addition to LDL-C, higher levels of ApoB and TG were found to be negatively associated with early AMD risk (Figs. 1D–E and 2). There is strong evidence for a causal association between TG and early AMD, based on IVW (*OR* = 0.77, 95% *CI*: 0.71–0.84, *P* = 5.02 × 10^−10^), weighted median (*OR* = 0.78, 95% *CI*: 0.68–0.88, *P* = 8.30 × 10^−5^), weighted mode (*OR* = 0.82, 95% *CI*: 0.73–0.92, *P* = 7.30 × 10^−4^), and MR Egger (*OR* = 0.79, 95% *CI*: 0.69–0.89, *P* = 2.14 × 10^−4^) approaches. Estimates from the IVW and weighted mode methods indicate that a higher ApoB level is significantly associated with decreased risk of early AMD (IVW: *OR* = 0.52, *P* = 6.60 × 10^−5^; weighted mode: *OR* = 0.56, *P* = 1.85 × 10^−3^), although these results were not significant after Bonferroni correction (Table 1).

High levels of CHOL were nominally associated with decreased risk of early AMD (Figs. 1F and 2, and Table 1). No associations were observed between Lp(a) and early AMD (Supplementary Fig. 1 and Table 1). To test whether there is reciprocal causation, we estimated the effects of early AMD on lipid biomarkers. As expected, the results showed no reciprocal causation (Supplementary Table 1).

### Sensitivity analysis

Using the MR-Egger intercept calculation, no evidence of directional horizontal pleiotropy was detected for any lipid-related traits (intercept was close to zero with *P*-value > 0.05). Results of MR-Egger intercept analysis were displayed in Table 1.

MR-PRESSO outlier-corrected tests were also performed in this study. MR analysis for all lipid-related traits showed that estimates and significance values were essentially unchanged after outlier SNPs were removed (Tables 2). These outlier SNPs were located primarily in genes that were associated with both lipid biomarkers and AMD (e.g., *CETP* and *APOE*); therefore, removing these SNPs would affect our estimated effect sizes and study power. Leave-one-out analysis was also applied to assess the robustness of our MR findings. Leave-one-out analysis was conducted by determining whether our estimates were driven or biased by single SNPs. Finally, no outliers were observed across all seven lipid-related traits and early AMD risk (Fig. S1–7).

**Table 2:** MR-PRESSO estimates of the associations between seven serum lipid biomarkers and early age-related macular degeneration

Exposures	MR-PRESSO methods	Causal estimate	*p*-value	Distortion *p*-value
**ApoA**	**Raw**	**0.71**	**9.91 × 10**^**−6**^	**0.64**
**ApoA**	**Outlier corrected**	**0.65**	**2.33 × 10**^**−4**^	**0.64**
**ApoB**	**Raw**	** − 0.65**	**1.00 × 10**^**−4**^	**0.45**
**ApoB**	**Outlier corrected**	** − 0.57**	**2.97 × 10**^**−4**^	**0.45**
**CHOL**	Raw	** − **0.13	7.38 × 10^**−**2^	0.43
**CHOL**	Outlier corrected	** − **0.18	2.23 × 10^**−**3^	0.43
**HDL-C**	**Raw**	**0.22**	**5.28 × 10**^**−8**^	**0.26**
**HDL-C**	**Outlier corrected**	**0.28**	**1.67 × 10**^**−12**^	**0.26**
**LDL-C**	**Raw**	** − 0.10**	**2.20 × 10**^**−3**^	**0.79**
**LDL-C**	**Outlier corrected**	** − 0.09**	**2.96 × 10**^**−3**^	**0.79**
**Lp(a)**	Raw	0.0004	1.92 × 10^**−**1^	NA
**Lp(a)**	Outlier corrected	NA	NA	NA
**TG**	**Raw**	** − 0.26**	**1.72 × 10**^**−9**^	**0.94**
**TG**	**Outlier corrected**	** − 0.26**	**2.24 × 10**^**−10**^	**0.94**

*P*-value < 0.0071 considered as significant at genome-wide association level and with bolded numbers

### Multivariable MR

We performed multivariable MR (MVMR)-IVW analysis to evaluate the direct effects of lipid biomarkers on early AMD by conditioning on other related traits. For the classic trio (HDL-C, LDL-C, and TG), we found strong evidence for associations between HDL-C (*OR* = 1.18, *P* = 0.0014) and TG (*OR* = 0.86, *P* = 0.0099) with the risk of early AMD. However, the association between LDL-C and early AMD was nominal (*P* = 0.063). Next, HDL-C was replaced with ApoA1 in the trio (ApoA, LDL-C, and TG). These results showed that ApoA (*OR* = 1.85, *P* = 0.00034) and TG (*OR* = 0.83, *P* = 0.00033) are significantly associated with risk of early AMD. For the group of ApoA, ApoB, TG, and Lp(a), the results were similar to those of univariable MR analysis (ApoA: *OR* = 1.58, *P* = 0.0060; ApoB: *OR* = 0.62, *P* = 0.0086; TG: *OR* = 0.83, *P* = 0.00017, Table 2).

## Discussion

In this study, we conducted comprehensive MR analyses to investigate the causal effects of seven serum lipid biomarkers on early AMD risk. We found that higher HDL-C and ApoA levels increase the risk of early AMD, whereas LDL-C, ApoB, and TG appear to be associated with decreased risk of early AMD. The major value of this study is to fill gaps in our understanding of the causal effects of lipid biomarkers on early AMD risk. More importantly, these MR findings shed light on the different roles of lipid subfractions in early AMD and aid us in understanding the role of lipid metabolism in the initiation and formation of drusen in early stages, as well as the potential utility of blood lipid-modifying therapies in preventing and treating AMD.

Compared with previous MR studies, the direction of causal effect size was the same as the causal effects of lipids on intermediate and late AMD, for which genetically elevated HDL-C levels increase AMD risk [17–19]. Han et al. found that high-density HDL-C and ApoA1 levels increase the risk of intermediate, GA, CNV, and advanced AMD subtypes [19]. In our study, the effect size of HDL-C levels on early AMD was 1.25 per 1 mmol/l increase (95% *CI*: 1.22–1.70), which appeared smaller than the effect on intermediate AMD (*OR* = 1.34 per 1 mmol/l increase, 95% *CI*: 1.20–1.49) [19]. Notably, we identified a robust association between HDL-C and early AMD, specifically using the inverse-variance weighted, weighted median, weighted mode, and MR Egger methods. Furthermore, our study also determined that higher levels of ApoA, the major apolipoprotein in HDL-C particles, increase the risk of early AMD, with *OR* 2.04 (95% *CI*: 1.50–2.77, *P* = 6.27 × 10^−6^, Table 2). Our ever-increasing knowledge of HDL-C highlights two sides of health. On the one hand, HDL-C induces reverse cholesterol transport associated with plaque regression and has anti-inflammatory [23] and antioxidant properties [24, 25], which could strengthen endothelial function and decrease atherosclerosis risk [26]. On the other hand, the adverse effects of high HDL-C levels on AMD may partially reflect HDL dysfunction. It has become apparent that under certain conditions, such as cardiovascular disease [27], aging [28], or acute phase response, HDL-C can play pro-inflammatory and pro-oxidant roles that inhibit cholesterol efflux [29, 30]. Consequently, oxidation products, such as peroxidation lipids, gradually accumulate in the retina and Bruch’s membrane, contributing to the development of AMD. Although a 12-month pilot study by Vavvas et al. reported that high-dose atorvastatin resulted in regression of drusen deposits in 10 of 23 patients [31], the impact of lipid-modifying therapies on AMD risk is currently unknown. Our results emphasize that the potential effects of lipid-modifying drugs on AMD and related phenotypes should be further investigated.

Consistent with previous observational studies, higher TG levels reduce the risk of early AMD [10, 12]. In our study, raised TG levels were associated with decreased risk of early AMD, with *OR* 0.77 (95% *CI*: 0.71–0.84, *P* = 5.02 × 10^−10^); the effect size was robust in MR-PRESSO outlier-corrected tests. In previous observational studies, LDL-C and CHOL levels were not found to be associated with any early AMD characteristics [12]. In MR studies, the causal association between LDL-C and advanced AMD was insignificant in both Europeans and Asians [17, 18]. However, Han X. et al. found that the associations of LDL-C were primarily with GA (*OR* = 0.70, 95% *CI*: 0.59–0.83, *P* = 3.8 × 10^−5^) and intermediate AMD (*OR* = 0.77, 95% *CI*: 0.67–0.87, *P* = 6.5 × 10^−5^) but were not strong enough with nAMD [19]. Similarly, ApoB (*OR* = 0.76, 95% *CI*: 0.69–0.85) and CHOL (*OR* = 0.81, 95% *CI*: 0.70–0.94) were also associated with decreased risk of intermediate AMD [19]. In our study, the lipid biomarkers LDL-C (*OR* = 0.90, 95% *CI*: 0.85–0.96, *P* = 2.03 × 10^−3^) and ApoB (*OR* = 0.52, 95% *CI*: 0.38–0.72, *P* = 6.60 × 10^−5^) play protective roles in the development of early AMD. However, we did not find robust associations between LDL-C and ApoB with early AMD in weighted median analyses, suggesting that the relationship between LDL-C and early AMD is inconsistent. In addition, we found no evidence of an association between Lp(a) and AMD risk.

Dyslipidemia has been implicated in the formation of drusen, which is characteristic of the early stage of AMD. Several lines of evidence support a role for dyslipidemia in AMD pathogenesis. First, histological evidence has identified that drusen are composed of lipid material [8, 32]. Second, animal experiments have demonstrated that impaired macrophage cholesterol efflux through HDL-mediated reverse cholesterol transport may lead to a pro-angiogenic status in AMD [33, 34]. Third, genetic association studies have identified risk variants in genes involved in lipid metabolism and in the transfer of lipids among lipoproteins, such as hepatic lipase C (LIPC), lipoprotein lipase (LPL), cholesterol ester transferase (CETP), ABC-binding cassettes A1 (ABCA1), and apolipoprotein E (APOE), all of which have been shown to influence the course of AMD pathobiology [35–37]. Moreover, previous MR studies have found the dyslipidemia is causal in the transition from intermediate to late AMD [17–19]. Additionally, this study emphasizes that serum lipid levels also have a causal effect on early AMD.

This study is mainly based on early AMD and provides additional insights into the causal roles of seven serum lipid biomarkers. A key strength of this study is that we used large-scale datasets with standard protocols to measure lipid biomarkers; this allowed us to systematically evaluate the effects of lipids on AMD risk. Compared with traditional studies, MR findings are less likely to be affected by confounding factors or biases associated with reverse causation. At the same time, our results should be interpreted in light of the limitations of the study. First, as this study was based on participants of European ancestry, the generalizability of our findings to other ethnic groups requires further investigation. Second, in the MR framework, genetically predisposed biomarker changes were assumed to have linear and lifetime effects on AMD risk. Therefore, the potential nonlinear relationships and short-term effects of these biomarkers remain unclear. Third, although biochemical evidence suggests that retina-specific lipid transport is facilitated by proteins regulating systemic lipid metabolism [38], retina-specific lipid concentrations would be more relevant measurements for understanding AMD risk. Further studies are needed to investigate the effects of retina-specific lipid metabolism on AMD risk. Furthermore, blood lipid levels change throughout life. Therefore, long-term follow-up studies are needed to understand the impact of dyslipidemia on AMD risk and progression over time. Finally, analysis of multiple variants does not point to a single mechanism being causal for AMD risk. Nevertheless, our study has important research and clinical implications. Our findings show that plasma HDL-C levels are directly connected to AMD pathogenesis, suggesting that modulating HDL-C metabolism may be a new strategy for delaying or preventing AMD. Although drugs that increase HDL-C levels, such as statins, are used to treat cardiovascular disease [39], their influence on AMD risk has not been reliably duplicated [40]. The inverse relationship between AMD and cardiovascular disease reflects a more complex role of lipid metabolism in the development of AMD. In addition, we did not observe a high genetic correlation between AMD and cardiovascular disease, consistent with previous findings [19]. As a result, lipid-modifying medications must be used with great caution, and additional research into the possible influence of HDL-raising medicines on AMD and its associated phenotypes is required.

## Conclusions

This study identifies causal relationships between elevated circulating HDL-C/ApoA levels and increased risk of early AMD, whereas TG specifically decreases the risk of early AMD. Our results also support further investigation into the use of lipid-modifying strategies to prevent or treat early AMD.

## Methods

### Study overview

This study used a two-sample MR approach to estimate the causal impact of exposures on the outcome using independent GWAS statistics. A total of seven lipid biomarkers, including apolipoprotein A (ApoA), apolipoprotein B (ApoB), total cholesterol (CHOL), high-density lipoprotein cholesterol (HDL-C), low-density lipoprotein cholesterol (LDL-C), lipoprotein A [Lp(a)], and triglycerides (TG), were used as exposures, and early AMD was the outcome. All data used in the current study is publicly available GWAS data, and the relevant ethical approvals can be found in the corresponding studies. Multiple testing was performed by Bonferroni correction, and significance was defined as a *P*-value < 0.0071 (= 0.05/7).

### Genetic instruments for serum lipid biomarkers

GWAS summary datasets for serum lipid biomarkers were obtained from the OpenGWAS database developed by the MRC Integrative Epidemiology Unit (IEU) at the University of Bristol (https://gwas.mrcieu.ac.uk/). Information on each lipid biomarker GWAS is summarized in Table 3. To ensure robust associations between genetic instruments and each lipid biomarker, we selected only genetic variants that reached genome-wide significance (*P* < 5 × 10^−8^) for MR analysis. If more than one GWAS dataset was available for a lipid biomarker, we selected the dataset containing the most valid genetic instruments.

**Table 3:** Description of the GWAS statistics of lipid biomarkers

Traits	GWAS accession number	Sample size	Number of SNPs	Population	Units
**ApoA**	ukb-d-30630_raw	313,387	13,585,234	European	g/L
**ApoB**	ukb-d-30640_raw	342,590	13,585,958	European	g/L
**CHOL**	met-d-Total_C	115,078	12,321,875	European	mmol/L
**HDL-C**	ieu-b-109	403,943	12,321,875	European	mmol/L
**LDL-C**	ebi-a-GCST90002412	431,167	16,293,344	European	mmol/L
**Lp(a)**	ukb-d-30790_raw	273,896	13,583,854	European	nmol/L
**TG**	ieu-b-111	441,016	12,321,875	European	mmol/L

### GWAS summary statistics for early AMD

GWAS summary data for early AMD was obtained from a recently published genome-wide association meta-analysis which successfully identified multiple novel loci [22]. Winkler and colleagues gathered GWAS data for early AMD from 11 sources, including the International AMD Genomics Consortium (IAMDGC) and UK Biobank (UKBB) [22]. In total, 14,034 cases and 91,214 controls were included in the analysis (number of SNPs = 11,304,110). All included individuals were of European ancestry.

### Statistical analysis

To ensure that only uncorrelated variants were included in MR analysis, all variants correlated with the most significant SNPs were excluded (clumping *r*^2^ cut-off = 0.001, clumping window = 10,000 kb). Subsequently, all included SNPs were extracted from the GWAS dataset for early AMD, and alleles were harmonized. MR analysis was conducted using the TwoSampleMR R package [41]. The inverse-variance-weighted (IVW) method was the primary analysis method used to assess the causal effect of each lipid biomarker on early AMD.

To assess the potential impact of horizontal pleiotropy, a series of sensitivity analyses were performed, including MR-Egger regression, weighted median method, and weighted mode method [41]. These methods allow for the existence of horizontal pleiotropy but have less statistical power than IVW [41]. Additionally, the Egger intercept was calculated to assess horizontal pleiotropy [42]. MR pleiotropy residual sum and outlier (MR-PRESSO) testing was applied to assess potential bias from outliers and to evaluate overall heterogeneity [43]. Leave-one-out analysis was also applied to assess the influences of particular SNPs [41].

To avoid collinearity, we performed multivariable MR (MVMR)-IVW analysis by selecting groups of exposures. By using genetic instruments associated with any of the included sets of exposures, MVMR analysis can estimate the influence of each exposure condition on the effects of SNPs related to the other exposures.

## Supplementary Information

**Additional file 1: Table S1.** Reciprocal causation.

**Additional file 2: Tables S2 to S6.**

**Additional file 3. Supplemental figures.**

## Data Availability

The dataset(s) supporting the conclusions of this article is(are) included within the article (and its additional file(s)).

## Abbreviations

AMD: Age-related macular degeneration
GA: Geographic atrophy
Anti-VEGF: Anti-vascular endothelial growth factor
MR: Mendelian randomization
HDL-C: High-density lipoprotein cholesterol
ApoA: Apolipoprotein A
ApoB: Apolipoprotein B
CHOL: Total cholesterol
LDL-C: Low-density lipoprotein cholesterol
Lp(a): Lipoprotein A
TG: Triglycerides
IAMDGC: International AMD Genomics Consortium
UKBB: UK Biobank
IVW: Inverse-variance weighted
MR-PRESSO: MR pleiotropy residual sum and outlier
MVMR: Multivariable MR
