# Supplementary material for: Causal effects of serum lipid biomarkers on early age-related macular degeneration using Mendelian randomization
Source: Genes Nutr. 2023 Jul 21;18:11. doi: 10.1186/s12263-023-00730-5 (PMC10362672; doi:10.1186/s12263-023-00730-5)
Supplement: Supplementary file 3 — Additional file 3. Supplemental figures. [file 12263_2023_730_MOESM3_ESM.pdf]

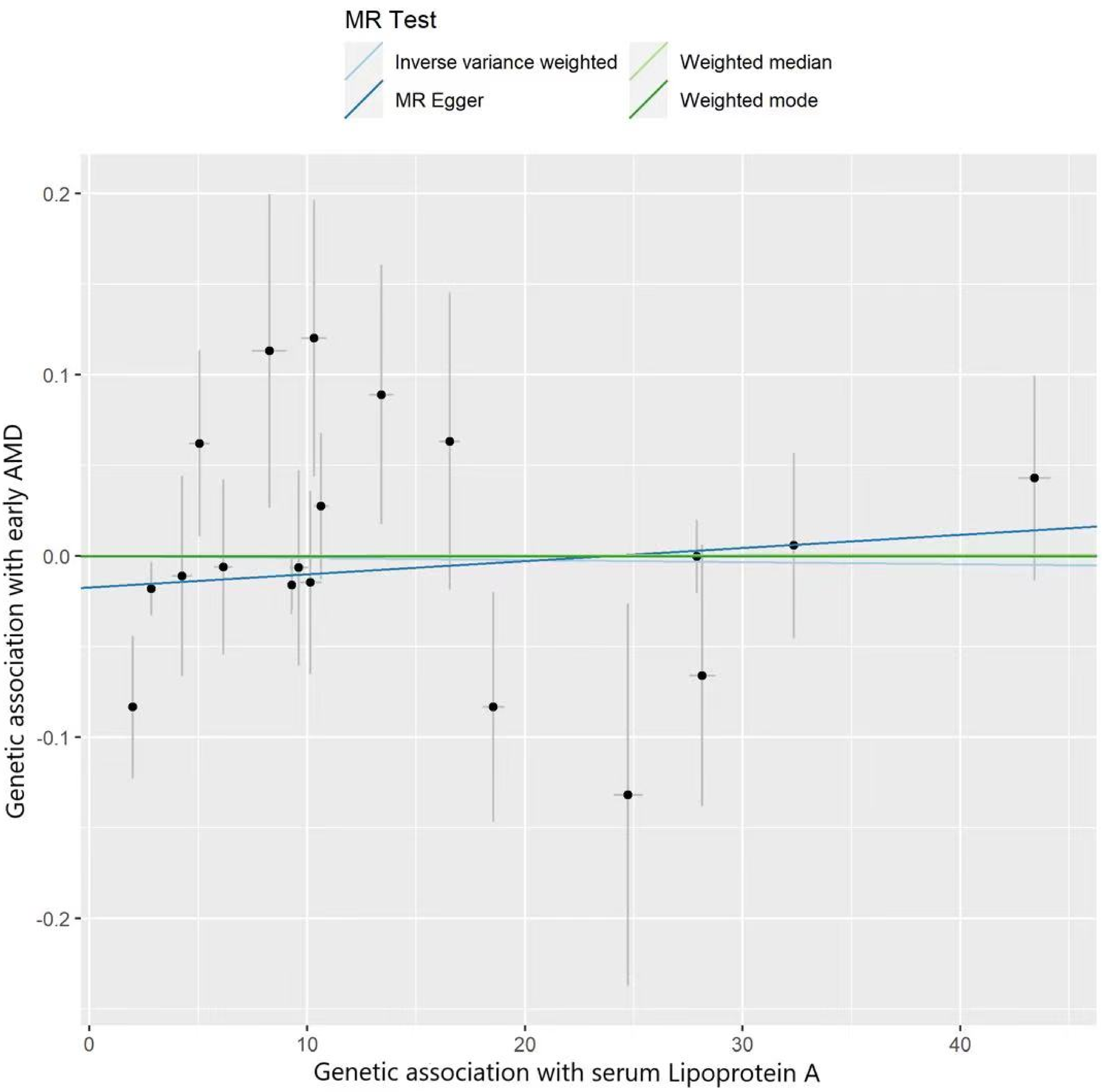
MR Test
Inverse variance weighted
Weighted median
MR Egger
Weighted mode
Genetic association with early AMD
0.2
0.1
0.0
-0.1
-0.2
0
10
20
30
40
Genetic association with serum Lipoprotein A

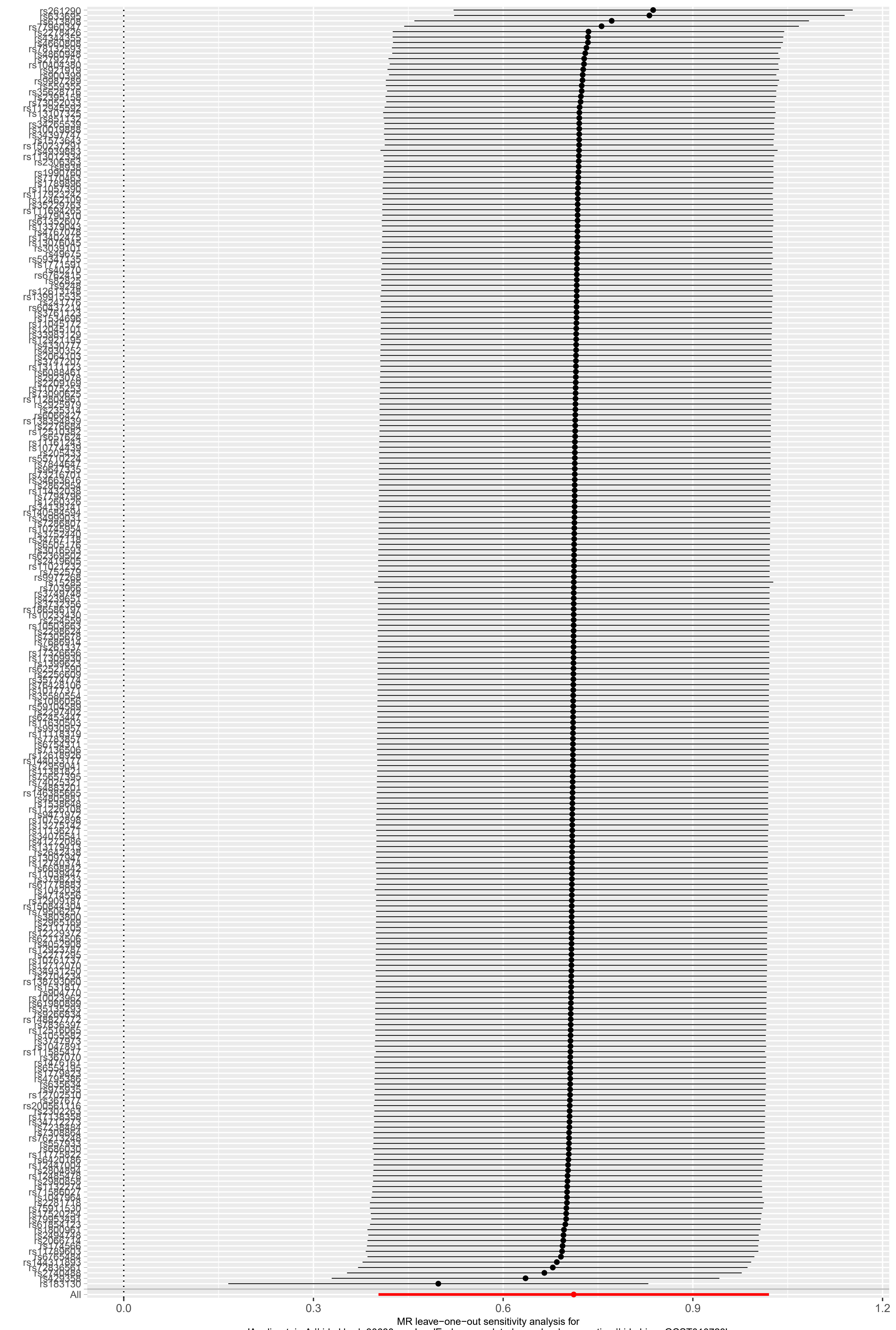

MR leave−one−out sensitivity analysis for
'Apoliprotein A || id:ukb-d-30630_raw' on 'Early age−related macular degeneration || id:ebi−a−GCST010723'

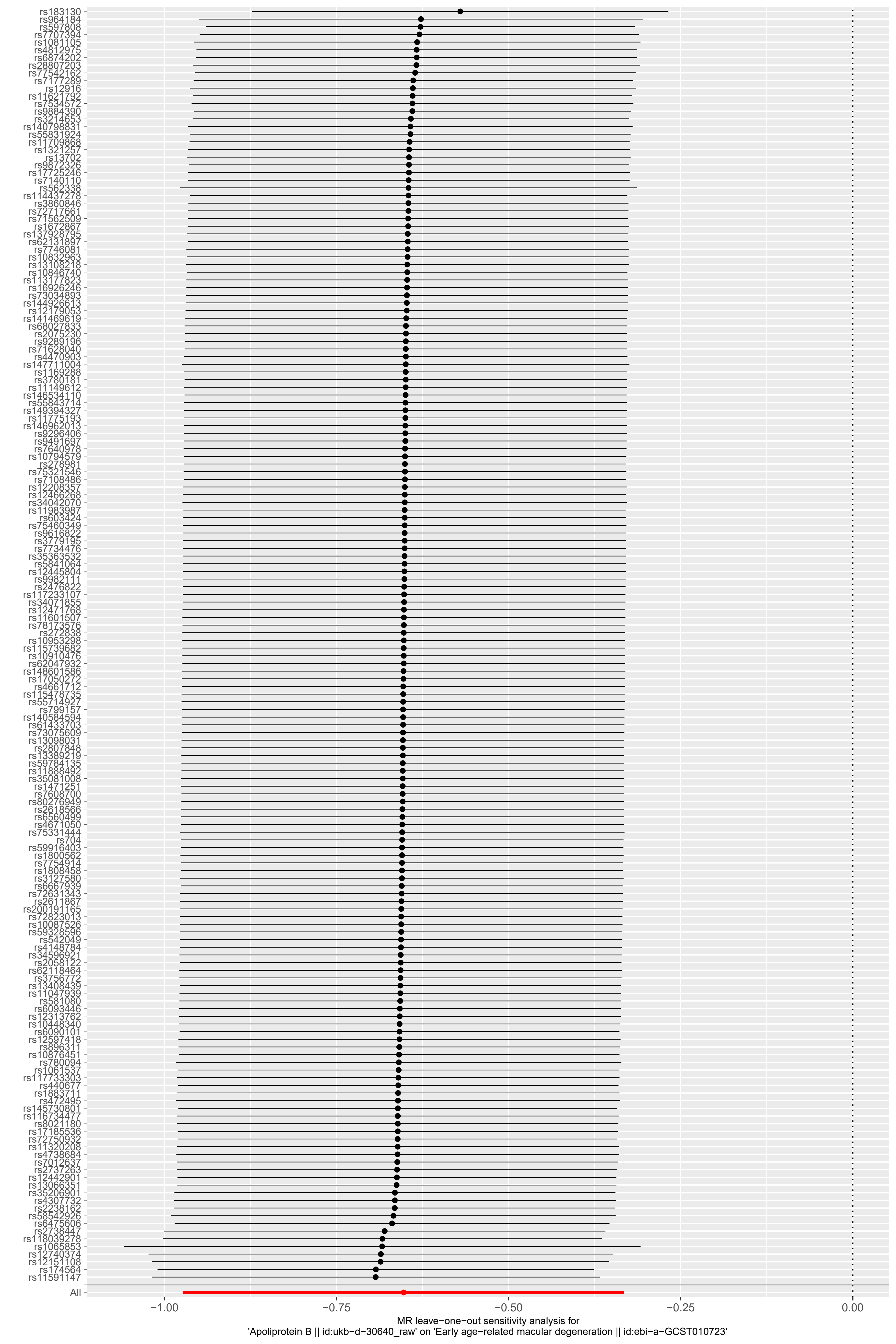
rs183130
rs964184
rs597808
rs7707394
rs1081105
rs4812975
rs6874202
rs28807203
rs77542162
rs7177289
rs12916
rs11621792
rs7534572
rs9884390
rs3214653
rs140798831
rs55831924
rs11709868
rs1321257
rs13702
rs9872326
rs17725246
rs7140110
rs562338
rs114437278
rs3860846
rs72717661
rs71562509
rs1672867
rs137928795
rs62131897
rs7746081
rs10832963
rs13108218
rs10846740
rs113177823
rs16926246
rs73034893
rs144926613
rs12179053
rs141469619
rs68027833
rs2075230
rs9289196
rs71628040
rs4470903
rs147711004
rs1169288
rs3780181
rs11149612
rs146534110
rs55843714
rs149394327
rs11775193
rs146962013
rs9296406
rs9491697
rs7640978
rs10794579
rs278981
rs75321546
rs7108486
rs12208357
rs12466268
rs34042070
rs11983987
rs603424
rs75460349
rs9616822
rs3779195
rs7734476
rs35363532
rs5841064
rs12445804
rs9982111
rs2476822
rs117233107
rs34071855
rs12471768
rs11601507
rs78173576
rs272838
rs10953298
rs115739682
rs10910476
rs62047932
rs148601586
rs17050272
rs4661712
rs115478735
rs55714927
rs799157
rs140584594
rs61433703
rs73075609
rs13098031
rs2807848
rs13389219
rs59784135
rs11888492
rs35081008
rs1471251
rs7608700
rs80276949
rs2618566
rs6560499
rs4671050
rs75331444
rs704
rs59916403
rs1800562
rs7754914
rs1808458
rs3127580
rs6667939
rs72631343
rs2611867
rs200191165
rs72823013
rs10087526
rs59328596
rs542049
rs4148784
rs34596921
rs2058122
rs62118464
rs3756772
rs13408439
rs11047939
rs581080
rs6093446
rs12313762
rs10448340
rs6090101
rs12597418
rs896311
rs10876451
rs780094
rs1061537
rs117733303
rs440677
rs1883711
rs472495
rs145730801
rs116734477
rs8021180
rs17185536
rs72750932
rs11320208
rs4738684
rs7012637
rs2737263
rs12442901
rs13066351
rs35206901
rs4307732
rs2238162
rs58542926
rs6475606
rs2738447
rs118039278
rs1065853
rs12740374
rs12151108
rs174564
rs11591147
All
−1.00
−0.75
−0.50
−0.25
0.00
MR leave−one−out sensitivity analysis for
'Apolipoprotein B || id:ukb−d−30640_raw' on 'Early age−related macular degeneration || id:ebi−a−GCST010723'

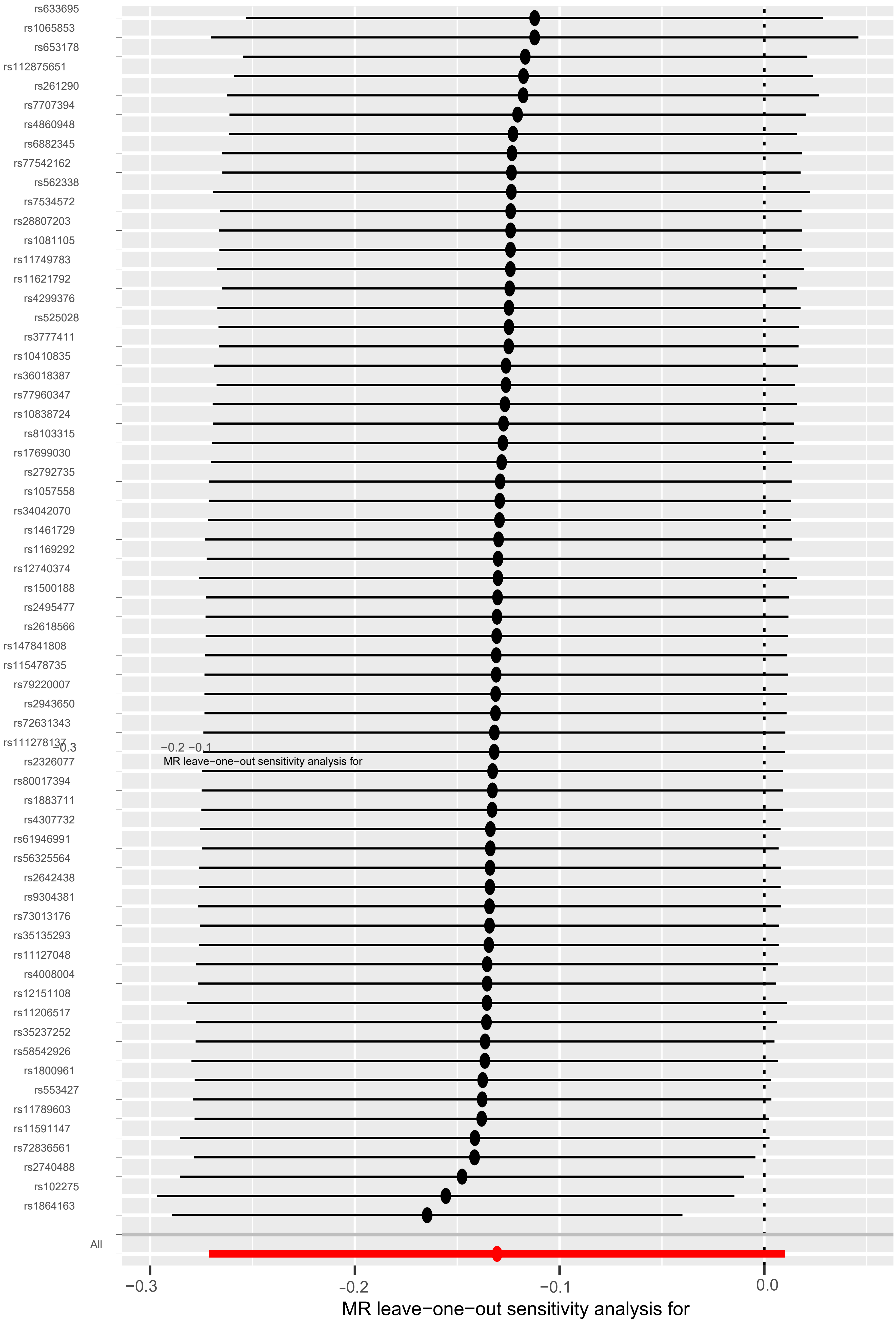

'Total cholesterol || id:met−d−Total_C' on 'Early age−related macular degeneration || id:ebi−a−GCST010723'

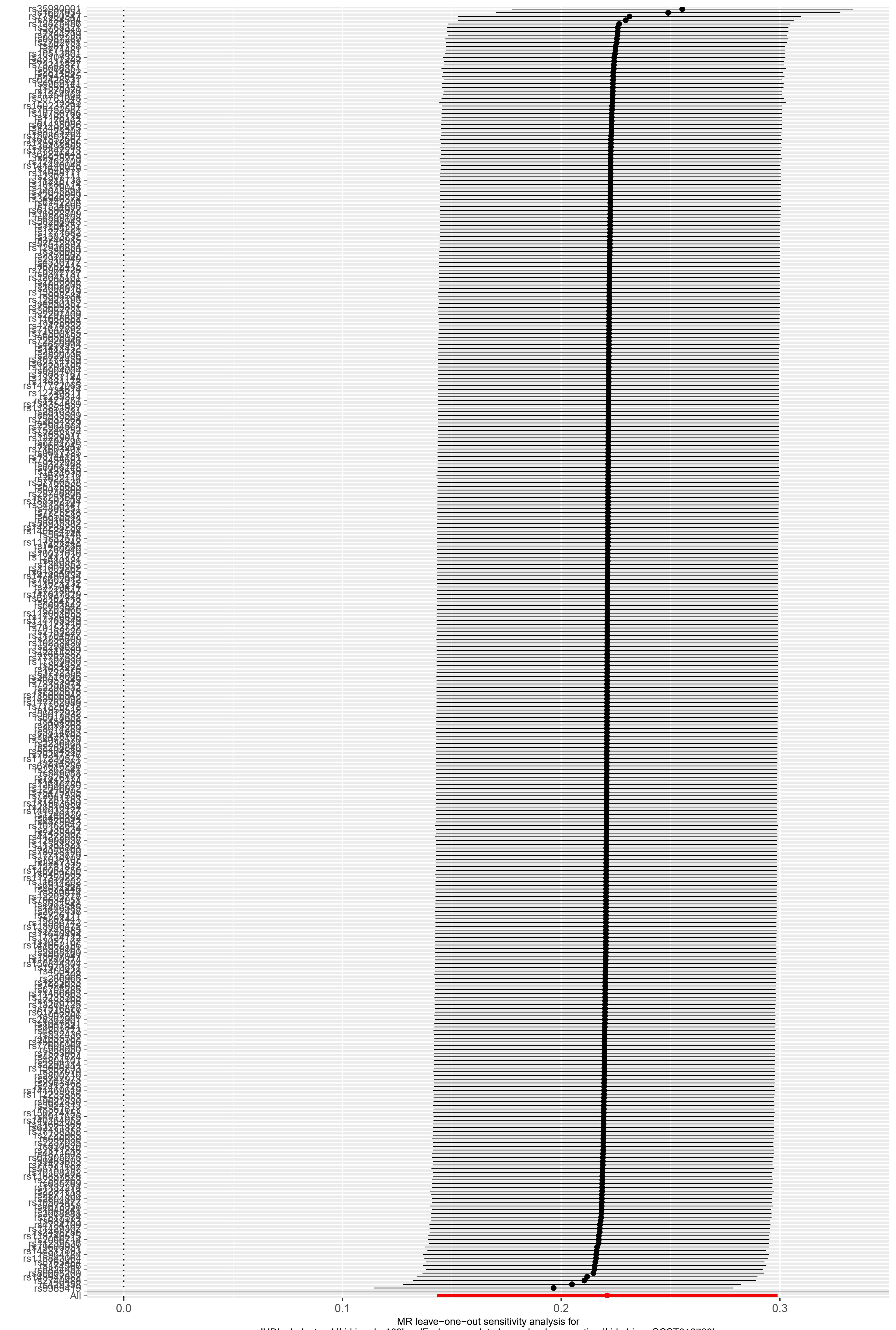

MR leave−one−out sensitivity analysis for
'HDL cholesterol || id:ieu−b−109' on 'Early age−related macular degeneration || id:ebi−a−GCST010723'

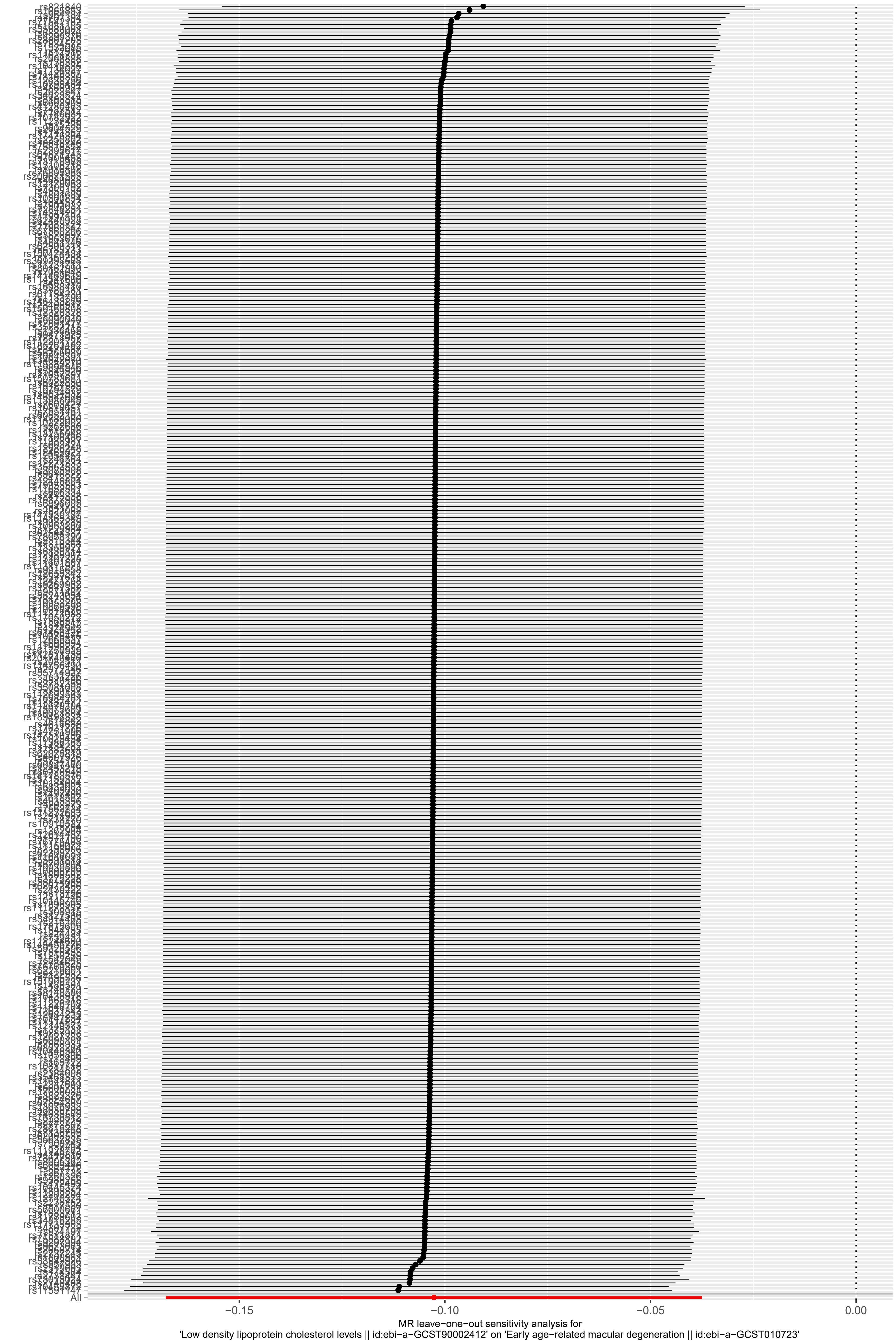
rs821840
rs11591147
All
−0.15
−0.10
−0.05
0.00
MR leave−one−out sensitivity analysis for
'Low density lipoprotein cholesterol levels || id:ebi−a−GCST90002412' on 'Early age−related macular degeneration || id:ebi−a−GCST010723'

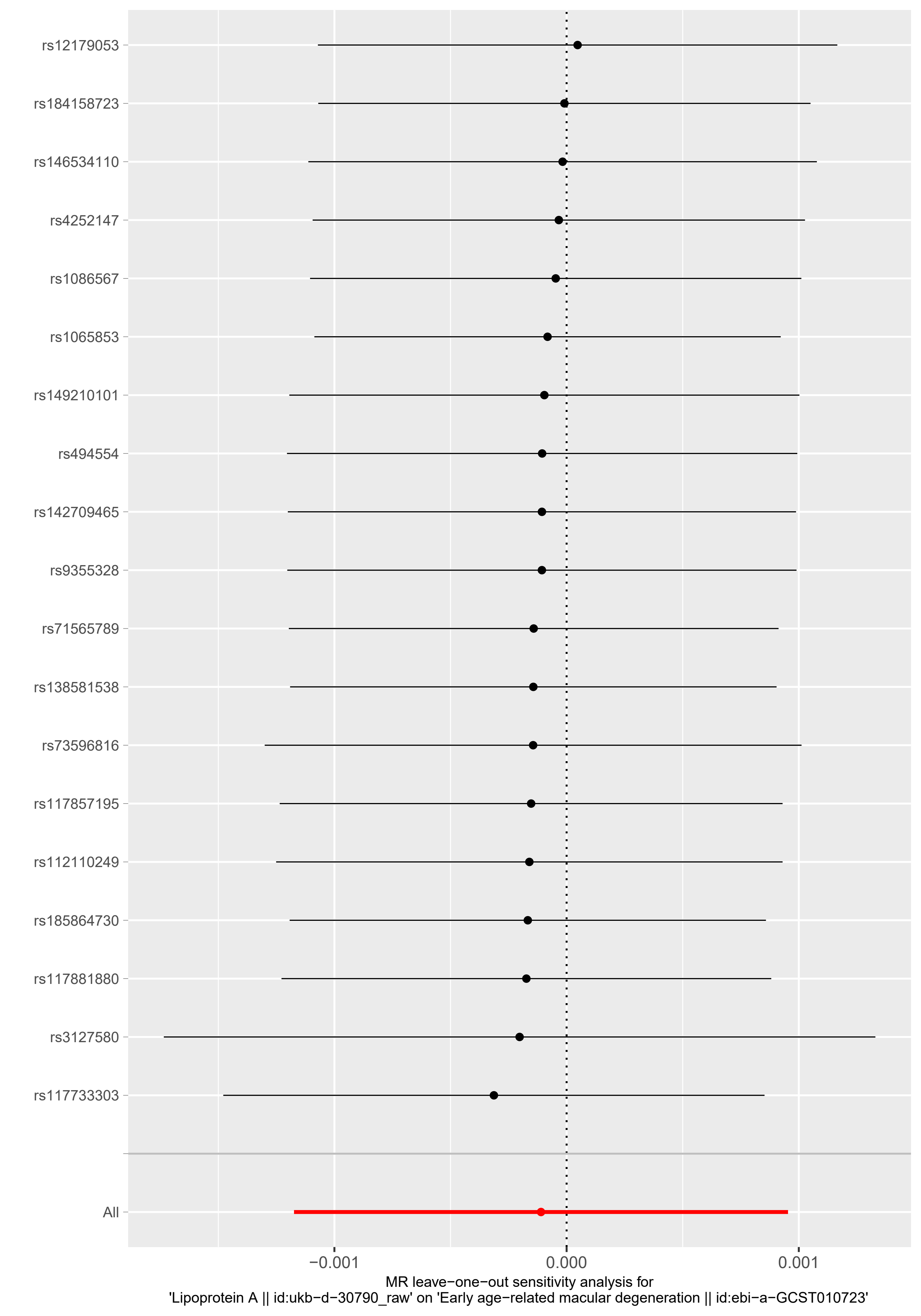

rs12179053
rs184158723
rs146534110
rs4252147
rs1086567
rs1065853
rs149210101
rs494554
rs142709465
rs9355328
rs71565789
rs138581538
rs73596816
rs117857195
rs112110249
rs185864730
rs117881880
rs3127580
rs117733303
All
−0.001
0.000
0.001
MR leave−one−out sensitivity analysis for
'Lipoprotein A || id:ukb−d−30790_raw' on 'Early age−related macular degeneration || id:ebi−a−GCST010723'

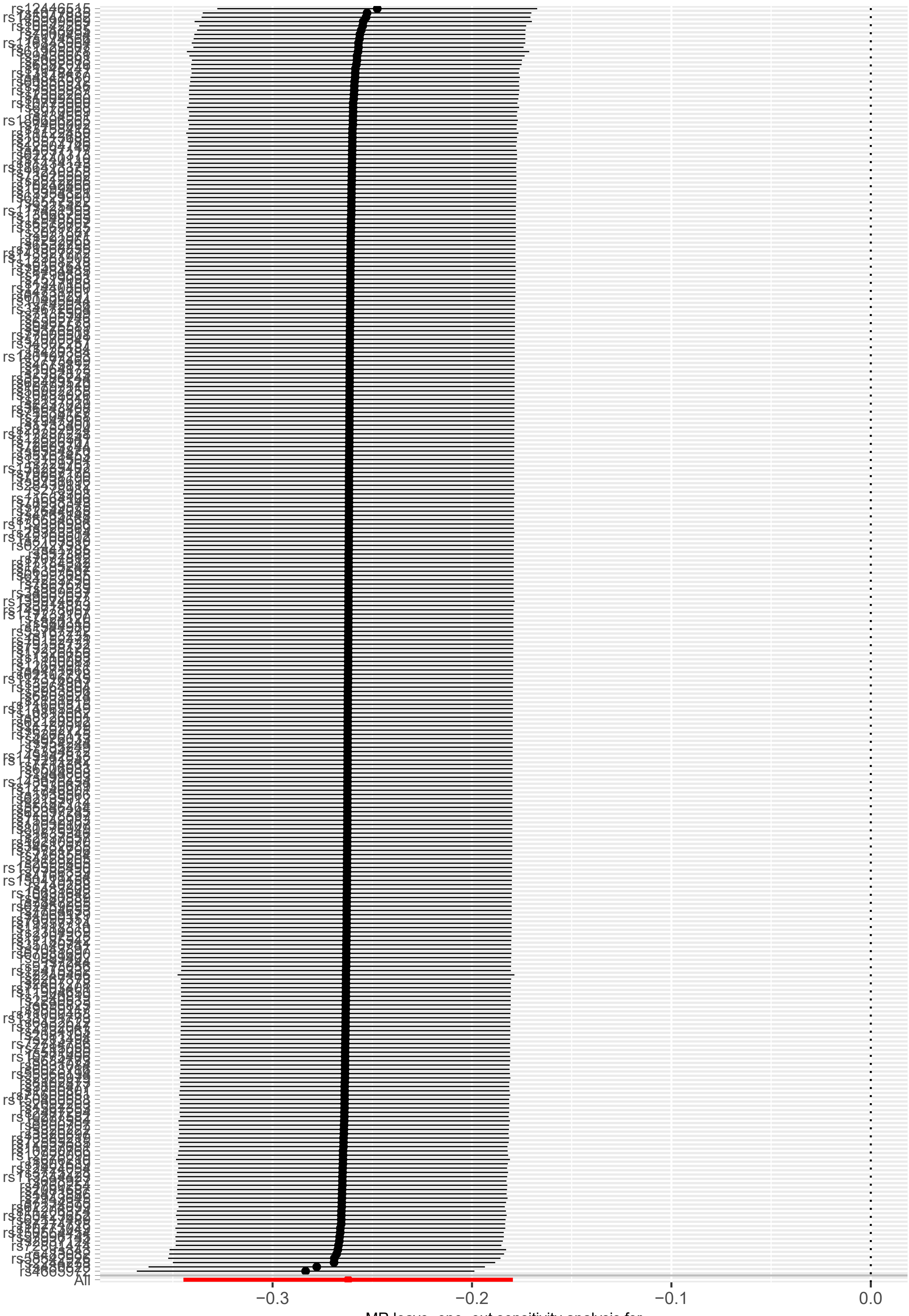

MR leave−one−out sensitivity analysis for
'triglycerides || id:ieu−b−111' on 'Early age−related macular degeneration || id:ebi−a−GCST010723'
